# Insights from a six‐year hair drug analysis compendium in drug‐facilitated crimes involving vulnerable population cases

**DOI:** 10.1111/1556-4029.70330

**Published:** 2026-04-16

**Authors:** Amandine Fort, Coralie Boudin, Hélène Eysseric‐Guerin, Virginie Scolan, François Paysant, Cloé Scherpereel, Mélissa Revet, Françoise Stanke‐Labesque, Théo Willeman

**Affiliations:** ^1^ Clinique de Médecine Légale CHU Grenoble Alpes Grenoble France; ^2^ Département de Médecine Légale Université Grenoble Alpes Grenoble France; ^3^ Laboratoire de Pharmacologie, Pharmacogénétique et Toxicologie CHU Grenoble Alpes Grenoble France; ^4^ Inserm 1300‐ HP2, CHU Grenoble Alpes, University Grenoble Alpes Grenoble France

**Keywords:** DFC, drug‐facilitated crime, forensic toxicology, hair analysis, mass spectrometry, MS, segmental analysis

## Abstract

Hair analysis is a well‐established matrix in forensic toxicology, offering a valuable alternative or complement to traditional matrices in diverse contexts, including drug‐facilitated crimes (DFC), elder abuse, and accidental exposure in children. However, objective interpretation of hair drug concentrations remains challenging due to significant inter‐study variability, even for identical compounds. This study included hair analysis results upon the prosecutor's request over six years in the Grenoble Forensic Laboratory in France. Hair analysis was performed using liquid chromatography coupled with tandem mass spectrometry (LC–MS/MS) on Sciex® 5500QT and Waters® TQ‐XS mass spectrometers, following the French Society of Analytical Toxicology (SFTA) guidelines. Hair proficiency quality testing ensured reproductive results. This compendium describes 38 cases with hair analysis, including child's exposure and chemical abuse involving both children and the elderly. The study population predominantly involved female victims (84.2%), aged 5 months to 90 years. At least one substance was detected in hair in 26 cases. In twelve cases, hair analyses supplemented blood or urine testing. Substances identified were cocaine, THC, and levamisole in child exposure cases; alprazolam, diazepam, loprazolam, and tiapride in chemical abuse; and mainly MDMA, cocaine, paroxetine, and THC in other cases. This compendium contributes valuable data to the literature, enhancing the interpretation of hair drug concentrations across a broad spectrum of cases characterized by diverse age ranges, hair characteristics, timeframes, and circumstances. This work provides critical comparative data for interpreting findings in forensic cases.

**Highlights**

- Authentic hair analyses contextualized for forensic interpretation.
- Cocaine and THC were the primary drugs in child exposure cases.
- Hair analysis aids the detection of repeated drug exposure in elder abuse.
- Hair analysis applies to diverse forensic cases: criminals, minors' risky behavior.
- Study enhances forensic interpretation of hair drug concentrations.

## 1 INTRODUCTION

Drug‐facilitated crimes (DFC) involve the administration of psychoactive substances to enable the commission of criminal acts. These offenses include drug‐facilitated sexual assault (DFSA), theft, and physical or psychological abuse, but may also extend to situations of neglect, such as accidental or unintended environmental exposure. A concerning upward trend has been reported in recent years. Notably, a 2022 survey conducted by the Paris Addictovigilance Center documented an annual increase of approximately 3% in suspected cases of chemical submission. This observation is consistent with the growing body of evidence indicating a rising prevalence of DFC worldwide [1].

It is important to distinguish these offenses from incidents involving chemical vulnerability or accidental toxic exposure [2]. In DFC, psychoactive substances are typically administered to incapacitate victims, rendering them unable to consent or resist acts of violence. However, these crimes also affect other vulnerable populations, potentially exacerbating existing vulnerabilities or serving objectives beyond simple incapacitation [3].

Infantile occult exposure to illicit drugs in domestic environments represents a complex clinical and medico‐legal challenge. Such exposure may be associated with abuse or neglect and can result in both short‐ and long‐term health consequences for children. Medicinal products acting on the central nervous system, including analgesics, anxiolytics, and antipsychotics, account for approximately 30% of drug‐related poisonings in pediatric populations. Cannabis exposure is responsible for about 7% of hospital admissions for poisoning in children under six years of age but accounts for up to 23% of poisoning‐related admissions to intensive care units within this age group [4]. Determining the route of contamination in these cases is particularly difficult, with possibilities including breastfeeding, environmental contamination, administration under duress or exploitation of vulnerability, or accidental ingestion [5, 6, 7, 8]. The variety of these situations, coupled with the physicochemical properties of children's hair, underscores the importance of documenting cases involving child abuse and accidental exposure.

Another vulnerable population that has received limited attention in the literature is the elderly. Intentional intoxication in this group is often aimed at facilitating theft, although cases of abuse have also been reported [9]. Benzodiazepines, neuroleptics, and opiates are suggested as the most frequently implicated substances [10].

In cases of delayed detection or repeated intoxication, hair analysis has emerged as a valuable forensic tool. It extends the detection window for xenobiotic exposure to several months, complementing conventional blood and urine analyses in toxicological investigations and providing a longer‐term perspective on substance exposure. However, limitations such as potential external contamination, low analyte concentrations, variable drug incorporation and hair growth, the impact of cosmetic treatments, and difficulties in data interpretation must be considered. Therefore, meticulous sample collection, validated analytical methods, and cautious interpretation of the results in conjunction with other toxicological and investigative findings are necessary. Furthermore, reference data for interpreting drug concentrations in hair are sometimes lacking in the scientific literature.

In this context, the present study aims to compile and analyze cases of DFC and familial exposures identified through hair analysis at Grenoble Alpes University Hospital from 2019 to 2024. This compendium seeks to contribute to the understanding and interpretation of forensic cases involving hair analysis, particularly within the context of chemical abuse in vulnerable populations and familial exposures.

## 2 MATERIALS AND METHODS

### 2.1 Case inclusion

Hair collection and analysis were performed upon prosecutor's request. Cases were included over 6 years from January 2019 to December 2024.

### 2.2 Hair collection

Hair collection was performed by a trained professional (a toxicologist or a forensic pathologist), at least 4 to 6 weeks after potential exposure. This interval allowed sufficient time for the hair segment formed during the exposure period to emerge from the scalp, given an average hair growth rate of approximately 1.0 cm per month, thereby enabling detection of incorporated substances in the proximal segment [11]. Sample collection was conducted in accordance with established good practice guidelines [12]. A lock of hair was isolated using a string and cut as close to the scalp as possible from the vertex posterior region, using scissors previously cleaned with an alcohol‐free wipe. When scalp hair was unavailable, hair from other body sites was collected. The collected lock was secured to a sheet of white paper by taping both ends of the string, ensuring that the adhesive did not come into contact with the hair. The proximal (root) end was clearly indicated. The hair sample was then placed in a labeled envelope before being shipped to the Grenoble Alpes University Hospital (CHUGA) Pharmacology, Pharmacogenetics, and Toxicology Laboratory. Samples were stored at room temperature upon analysis.

### 2.3 Preparation of hair samples

Each lock of hair was described with the following parameters: color, total length and weight with and without the string. The orientation of hair samples was preserved thanks to the string at the proximal end of the lock of hair. External decontamination was performed by washing the hair samples twice in dichloromethane. In each step, samples were placed in a beaker and manually agitated for 1 min before being blotted dry with absorbent paper. Hair alignment was verified prior to and following the decontamination procedure. The portion to be analyzed was cut into several segments when it was possible. Segments were then finely chiseled into pieces of less than 2 mm and homogenized. Analyses were carried out on test samples ranging from 10 to 20 mg; concentrations were normalized based on the ratio between the actual hair mass analyzed and the mass used for the calibration standards. Sample preparation was identical for all analytical methods and has been previously described in detail [13]. 20 mg of hair were fortified with 10 μL of internal standard at 0.1 mg/L for psychotropic medication, 4 μL at 1.0 mg/L for amphetamines, opiates, cocaine (AOC), and 4 μL at 0.1 mg/L for cannabinoids. After the addition of 400 μL of MeOH, samples were placed in an ultrasonic wash for 10 min and then incubated overnight at 55°C for AOC and cannabinoids, and at ambient temperature for psychotropic medication. Following centrifugation for 20 min at 25,000 rpm, the supernatant was evaporated to dryness under a gentle stream of nitrogen and reconstituted with 200 μL of mobile phase A prior to chromatographic analyses.

### 2.4 Analytical toxicology procedures

Following SFTA guidelines [14], several analytical methods were carried out depending on the context and prosecutor's request (Table 2). Both hair extracts and decontamination wash solutions were analyzed to assess and document the possibility of external contamination.

#### 2.4.1 Quantitative LC–MS/MS methods for illicit and sedative drugs

Four quantitative methods were carried out on the different segments.

Firstly, a targeted method of benzodiazepines and related products (29 anxiolytics and/or hypnotic molecules) and secondly a targeted method of sedative psychotropic medication (24 molecules neuroleptics and/or anti‐histamines) were performed. A methanolic extract obtained after incubating the segments for 12 h in methanol at room temperature was analyzed using LC–MS/MS on a Kinetex® column (XB‐C18 100 Å; 50 × 2.1 mm; Phenomenex, 2.6 μm). A comprehensive description of the chromatographic conditions and MS parameters is provided in Table S1.

Then, a method for AOC and metabolites and lastly a method for cannabinoids were performed, as previously described [15], on a LC system (Shimadzu, Kyoto, Japan) consisting of two LC‐20AD quaternary pumps and two LC‐20AD XR quaternary pumps equipped with a SIL‐20AC XR autosampler and a CTO‐20AC column compartment. Cannabinoids separation was performed with an online sample purification on a pentafluorophenyl (PFP) Kinetex column 5 μm × 2.1 mm × 50 mm (Phenomenex Aschaffenburg, Germany) and a chromatographic separation was performed on an XB C18 analytical column Kinetex 5 μm × 2.1 mm × 50 mm (Phenomenex, Aschaffenburg, Germany). AOC and metabolites separation were managed on a single biphenyl column Kinetex 3 μm × 2.1 mm × 100 mm (Phenomenex, Aschaffenburg, Germany).

Detection for the four methods was performed on an API 5500 tandem mass spectrometer (Sciex, Toronto, Canada) equipped with a Turbo Ion Spray® source. Quantification was achieved in the multiple reaction monitoring (MRM) mode, with one transition for quantification and one transition for confirmation per analyte and one ion transition per internal standard.

#### 2.4.2 Screening LC–MS/MS methods for medications and NPS

Two qualitative screening methods were also performed as already described [16]. The acquisition consisted of 2 scheduled targeted screenings containing 349 drugs and 166 new psychoactive substances (NPS), respectively. Ultrahigh performance liquid chromatography (UPLC) was performed on an I‐class Acquity system (Waters Milford, USA). Chromatographic separation was achieved using an Acquity HSS T3 column (100 mm x 2.1 mm, 2.5 μm) (Waters). Qualitative targeted screening was performed on a Xevo TQ‐XS (Waters).

#### 2.4.3 Methods validation

A blank hair sample was tested in each batch to ensure no contamination has occurred.

Internal quality controls were conducted for a series of assays, encompassing both commercial IQCs specific to our laboratory and three in‐house IQCs using spiked drug‐free head hair.

Accuracy, precision, lower limits detection (LOD) and lower limits of quantification (LOQ) are reported in Supplemental Information (Table S2).

Hair proficiency quality testing ensured reproductive results, with programs such as Arvecon DHF or French Society of Analytical Toxicology SOUCHI with satisfactory results.

### 2.5 Ethics

This study concerns forensic toxicological analysis at the request of the law enforcement authorities or a magistrate. Because this study used only routinely collected, de‐identified data, it did not require any ethics committee approval, and informed consent was waived in accordance with French regulations for mandatory reporting by health care professionals (Article R5132‐102 of the Public Health Code).

## 3 RESULTS

### 3.1 Population description

Between 2019 and 2022, a total of 38 cases involving hair analyses were identified. These included investigations of child exposure, chemical abuse affecting both children and elderly individuals, as well as other forensic cases arising from diverse circumstances. The demographic characteristics of the study population are summarized in Table 1. At least one substance was detected through hair analysis in 26 cases. Among these positive cases, blood and urine samples were also available in 12 instances and were generally within 24 h of the last suspected exposure.

**TABLE 1:** Population characteristics.

Variable (*N* = 38)		Percentage (%*N*)
*Sociodemographic*		
Mean age (age range)	26 y.o. (5 months–90 y.o.)	
Sex		
Female, *n*	32	84.2%
Male, *n*	6	15.8%
Status		
Victim, *n*	29	76.3%
Perpetrator, *n*	8	21.1%
Minor with risky behavior, *n*	1	2.6%
Positives cases	26	68.4%
*Victims' contexts*		
Location		
Home, *n*	13	72.2%
Public place, *n*	5	27.8%
*Missing data*	*11*	
Forensic situation		
Child exposure, *n*	7	53.8%
Chemical abuse, *n*	6	46.2%
*Children, n*	*3*	*50.0%*
*Elderly, n*	*3*	*50.0%*
*Missing data*	*16*	
*Hair samples characteristics*		
Hair type		
Scalp, *n*	37	97.4%
Other, *n*	1	2.6%
Mean sample's weight (min‐max) (mg)	649 (27.9–2343)	
Hair color		
Brown, *n*	23	60.5%
Dark, *n*	8	21.1%
Light, *n*	7	18.4%
Hair treatment		
Participants without hair bleaching, *n*	27	71.1%
Participant with hair bleaching, *n*	11	28.9%

*Note*: Values in italics indicate “missing data”.

The scope of toxicological analyses varied across cases, depending on the specific requirements outlined in the judicial requisitions (Table 2).

**TABLE 2:** Scope of toxicological analyses performed according to judicial requisitions.

Case	Matrice	BZD and related products	Sedative psychotropic medication	AOC and metabolites	Cannabinoid	NPS
*Child's exposure*						
1.1	Hair	No	No	Yes (JR)	No	No
	Blood & urine	Yes (JR)	Yes (JR)	Yes (JR)	Yes (JR)	Yes (JR)
1.2	Hair	No	No	Yes (JR)	Yes (JR)	No
	Blood & urine	No	No	Yes (JR)	Yes (JR)	No
2.1	Hair	Yes (JR)	Yes (JR)	Yes (JR)	Yes (JR)	Yes (JR)
	Urine	Yes (JR)	Yes (JR)	Yes (JR)	Yes (JR)	Yes (JR)
2.2	Hair	No	No	Yes (JR)	No	No
2.3	Hair	No	No	Yes (JR)	No	No
3.1	Hair	No	No	Yes (JR)	Yes (JR)	No
	Blood & urine	Unknown	Unknown	Yes (NR)	Unknown	Unknown
3.2	Hair	Yes (JR)	Yes (JR)	Yes (JR)	Yes (JR)	No
	Blood & urine	Yes (JR)	Yes (JR)	Yes (JR)	Yes (JR)	Yes (JR)
4.1	Hair	No	No	Yes (JR)	Yes (JR)	No
4.2	Hair	No	No	Yes (JR)	Yes (JR)	No
4.3	Hair	No	No	Yes (JR)	Yes (JR)	No
4.4	Hair	No	No	Yes (JR)	Yes (JR)	No
5	Hair	Yes (JR)	Yes (JR)	Yes (JR)	Yes (JR)	No
6	Hair					
	Blood & urine	Yes (JR)	Yes (JR)	Yes (JR)	Yes (JR)	Yes (JR)
*Abuse in children*						
1.1	Hair	Yes (JR)	Yes (JR)	No	No	No
1.2	Hair	Yes (JR)	Yes (JR)	No	No	No
2	Hair	Yes (JR)	Yes (JR)	No	No	No
	Urine	Yes (NR)	Yes (NR)	Unknown	Unknown	Unknown
*Abuse in the elderly*						
3	Hair	Yes (JR)	Yes (JR)	Yes (JR)	Yes (JR)	Yes (JR)
	Blood & urine	Yes (JR)	Yes (JR)	Yes (JR)	Yes (JR)	Yes (JR)
4	Hair	Yes (JR)	Yes (JR)	Yes (JR)	Yes (JR)	No
	Blood & urine	Yes (Unknown)	Yes (Uknown)	Unknown	Unknown	Unknown
5	Hair	Yes (JR)	Yes (JR)	Yes (JR)	Yes (JR)	No
*Other cases*						
1	Hair	Yes (NR)	Yes (NR)	Yes (JR)	No because insufficient sample size (JR)	No
2	Hair	Yes (JR)	Yes (JR)	Yes (JR)	Yes (JR)	No
3	Hair	Yes (JR)	Yes (JR)	Yes (JR)	Yes (JR)	Yes (JR)
	Urine	Yes (JR)	Yes (JR)	Yes (JR)	Yes (JR)	Yes (JR)
4	Hair	Yes (JR)	Yes (JR)	Yes (JR)	Yes (JR)	Yes (JR)
	Blood & urine	Yes (JR)	Yes (JR)	Yes (JR)	Yes (JR)	Yes (JR)
5	Hair	Yes (JR)	Yes (JR)	Yes (NR)	No	No
6	Hair	Yes (JR)	Yes (JR)	Yes (JR)	Yes (JR)	No
	Blood & urine	Yes (JR)	Yes (JR)	Yes (JR)	Yes (JR)	Yes (JR)
7	Hair	Yes (NR)	Yes (NR)	Yes (JR)	Yes (JR)	No

Abbreviations: AOC, amphetamines, opiates, cocaine; BZD, benzodiazepines; GBH, gamma‐hydroxybutyrate; No, analysis not performed; NPS, new psychoactive substances; Yes (JR), analysis performed following a judicial request; Yes (NR), analysis performed without a judicial request.

The substances most frequently identified differed according to case category: cocaine and THC were predominant in cases of child exposure, alprazolam was most commonly detected in chemical abuse cases, and MDMA and cocaine were prevalent in other miscellaneous forensic investigations.

### 3.2 Cases of child's exposure to narcotics

Thirteen cases of substance exposure in children were identified, including four family‐related cases and two isolated incidents. The toxicological findings for these cases are summarized in Table 3.

**TABLE 3:** Cases of child's exposure to narcotics.

Case	Status	Sex	Age (years)	Symptoms	Hair analysis				Substance	Segment length (cm) and hair concentrations (pg/mg) from root to tip	Other positive matrices	
					Time between last exposure and hair sampling (days)	Color	Hair bleaching	Number of analyzed segments (*n*/total)			Blood (ng/mL)	Urine (ng/mL)
1.1	Child	F	<1	None	210	Brown	No	10/10	Segment length	1.05–1.10–1.12–1.05–1.08–1.12–1.03^a^–1.08–1.13–0.94	BZE (Traces)	Cocaine (116)
									Cocaine	1700–1930–2610–3500–4540–5340–5400^a^–6180–6630–4180	Acetaminophen (nm)	BZE (321)
									BZE	397–450–759–991–1560–2216–2110^a^–2880–2477–2814		Acetaminophen (nq)
												Nicotine (nq)
												Lidocaine (nq)
1.2	Suspected consumer (mother)	F	32	–	–	Brown	No	18/18	Segment lenght	1.20–1.04–1.05–1.06–1.10–1.05–1.20–1.20–1.20–1.20–1.50–1.50–1.50–1.50^b^–2.00^b^–2.00^b^–2.00^b^–1.80^b^	na	BZE (ND)
									Cocaine	971–1371–1285–1601–594–726–645–564–732–1823–917–975–829–1145–2, 781^b^–1491^b^–1731^b^–2730^b^		THC‐COOH (9.4)
									BZE	462–197–128–79–58–67–72–85–106–129–85–84–86–111^b^–140^b^–112^b^–137^b^–221^b^		
									THC	343–789–739–664–683–640–687–615–607–552–474–356–209		
									CBN	99–214–311–540–315–567–365–476–408–476–381–373–358		
									CBD	167–344–367–475–394–423–411–419–371–376–340–321–286		
2.1	Child	F	3	None	45	Light	No	14/14	Segment length	1.06–0.98^a^–1.03–1.11–1.05–1.01–2.01–1.91–2.10–2.00–1.98–1.97–2.02–2.04	na	Cocaine (ND)
									Cocaine	70–180^a^–390–400–640–610–770–1080–900–890–1610–1310–1200–1530		BZE (47)
									BZE	ND–90^a^–60–110–220–210–250–250–150–140–250–210–390–520		EME (23)
2.2	Suspected consumer (aunt)	F	36	–	–	Brown with henna dyeing	Yes	10/10	Segment length	2.0–2.0–2.0–2.0–2.0–2.0–2.0–1.8–2.0–2.0	na	na
									Cocaine	14,400–18,600–22,600–21,200–20,100–26,700–25,600–22,100–25,500–27,100		
									BZE	4130–4730–5480–5770–5730–6550–6550–6470–6120–6050		
									EME	1340–1310–1980–1830–1590–1810–1940–1900–2110–2360		
									Cocaethylene	110–120–110–100–70–60–60–50–50–40		
2.3	Suspected consumer (mother)	F	34	–	–	Brown	No	13/13	Segment length	2.0–2.0–1.9–2.0–2.0–2.0–2.0–2.0–2.0–2.0–2.0–2.0–8.0	na	na
									Cocaine	20–30–50–70–110–130–140–110–90–70–100–120–220		
									BZE	ND–ND–ND–ND–20–20–30–20–10–10–20–20 40		
									EME	ND in all segments		
									Coca ethylene	ND in all segments		
3.1	Child	F	2	None	60	Brown	No	8/10	Segment length	3.0^a^–2.0–2.0–2.0–2.0–2.0–2.0–2.0	Cocaine (nq)	Cocaine (nq)
									Cocaine	269^a^–934–2693–2920–2611–3390–3225–2862		
									BZE	67^a^–242–668–821–927–1338–1426–1184		
									Norcocaine	Identified in all segments		
									Levamisole	Identified in all segments		
3.2	Suspected consumer (mother)	F	35	–	–	Brown	No	3/3	SEGMENT LENGTH	2.0–2.0–2.0	Ethylglucoronide (nq)	Ethylglucoronide (nq)
									COCAINE	751–1740–1840	Cotinine (nq)	Cotinine (nq)
									BZE	86–185–199		
									THC	205–564–421		
									Levamisole	Identified in all segments		
4.1	Child	M	<1	Unknown	Unknown	Brown	No	6/6	Segment length	1.0–1.0–1.0–1.0–1.0–2.0^d^	na	na
									THC	25–22–40–71–83–78^d^		
									CBD	14–13–20–43–48–37^d^		
4.2	Child	F	4	Unknown	Unknown	Brown	No	8/8 (+20 cm of unsegmented and unanalyzed tip)	Segment length	1.0–2.0–2.0–2.0–2.0–2.0–2.0–2.0	na	na
									THC	ND–ND–ND–14–20–25–29–34		
4.3	Suspected consumer (mother)	F	38	–	–	Brown	Yes	11/11 (+10 cm of unsegmented and unanalyzed tip)	Segment length	1.0–2.0–2.0–2.0–2.0–2.0–2.0–2.0–2.0–2.0–2.0	na	na
									THC	ND–21–82 ‐ 132–133–190–380–472–427–298–203		
									CBD	ND–10–44–62–90–124 – 163–186–176–146–108		
4.4	Suspected consumer (father)	M	47	–	–	Brownc^c^	No	1/1	Segment length	3.0	na	na
									THC	21		
5	Child	F	<1	Unknown	150	Brown	No	3/3	Segment length	2.0–2.1^a^–3.5	na	na
									THC	7–47^a^–43		
									Cocaine	734–2100^a^–3959		
									BZE	111–415^a^ 909		
6	Child	M	12	Unknown	Unknown	Dark	No	2/2	Segment length	2.0–2.5	THC (27.4)	THC (nq)
									Cocaine	ND–35	THC‐OH (4.5)	
									THC	82–226	THC‐COOH (64.7)	
									THC‐COOH	1.1–1.8		
									CBD	61–181		

Abbreviations: na, not analyzed; ND, not detected; nq, not quantified.

^a^ Segment corresponding to the last contact with the suspected consumer.

^b^ Pregnant period.

^c^ Axillary hair.

^d^ In‐utero exposure.

#### 3.2.1 Cases 1.1 and 1.2

A 9‐month‐old girl presented to the emergency department by her parents for evaluation of otitis. Cocaine was detected in her blood and urine. Hair analysis, conducted seven days post‐initial biological sampling (and last known exposure), revealed cocaine and its metabolite, BZE, in two hair segments. Hair samples from the mother also showed consistent concentrations of cocaine and its metabolite across all segments. Additionally, THC, CBN, and CBD were uniformly detected in all maternal hair segments, including those corresponding to pregnancy.

#### 3.2.2 Cases 2.1 to 2.3

A 3‐year‐old child, who had been placed in the care of her aunt, was referred to the emergency department after traumatic lesions were observed by nursery staff while the child was temporarily under her mother's supervision. The child's medical history was notable for a neurodevelopmental disorder of undetermined etiology, characterized by microcephaly, delayed motor development, behavioral disturbances with persistent agitation, episodes of self‐injurious behavior, and reduced pain sensitivity. Initial toxicological screening performed in the emergency department revealed the presence of cocaine metabolites in the child's urine. Hair analysis, performed 45 days after the suspected exposure, showed detectable concentrations of cocaine and its metabolites, with a decreasing concentration profile consistent with cessation of exposure. Notably, the concentrations of cocaine and BZE measured in the child's hair were higher than those observed in the mother's hair samples. In contrast, hair samples collected from the aunt revealed markedly elevated concentrations of cocaine, BZE, EME, and cocaethylene. These findings strongly suggested the aunt as the primary source of the child's exposure.

#### 3.2.3 Cases 3.1 and 3.2

A 2‐year‐old child, who was asymptomatic but for whom exposure was suspected and reported by her uncle, tested positive for cocaine in urine. Subsequent hair analysis, performed two months after biological sampling and last known contact, confirmed the presence of cocaine across all hair segments. A marked decrease in concentrations was observed from the segment corresponding to the most recent contact. The child's mother's hair revealed elevated cocaine levels, indicative of chronic use. Levamisole, a known adulterant in cocaine, was detected in both the child's and mother's hair, though not quantified.

#### 3.2.4 Cases 4.1 to 4.4

Hair samples from two children (6 months and 4 years) and their parents (all with brown hair except the mother with bleached hair) were analyzed for substance exposure. THC was detected in all hair segments of both children, with concentrations increasing from the root to the tip. CBD was additionally identified in the infant's hair. The mother's hair contained THC, with concentrations increasing from root to tip. For the father, only axillary hair was available for analysis. A single axillary hair segment showed a THC concentration of 21 pg/mg.

#### 3.2.5 Case 5

An 11‐month‐old child in foster care, with weekly visits from her biological mother, had THC, cocaine, and BZE detected in her brown hair samples, with concentrations increasing from root to tip. Six months prior, the mother's blood and urine were positive for THC and its metabolites (THC‐OH and THC‐COOH), while cocaine and its metabolites (BZE and EME) were detected only in her urine.

#### 3.2.6 Case 6

Hair samples were collected from a 12‐year‐old child who had been placed in a children's home. Urine toxicological screening revealed the presence of cannabis, indicating recent use (within less than one hour) as well as evidence consistent with regular cannabis consumption. These findings were complemented by hair analysis, which detected cocaine exclusively in the distal (tip) segment, at concentrations consistent with past passive environmental exposure. This exposure corresponded to a period during which the child was living with his father. THC, THC‐COOH, and CBD were detected in all hair segments.

### 3.3 Cases of chemical abuse involving both children and the elderly

Six cases of chemical abuse were identified, involving three children and three elderly subjects. The results are summarized in Table 4.

**TABLE 4:** Cases of chemical abuse involving both children and the elderly.

Case	Sex	Age (years)	Suspected vector	Symptoms	Hair analysis				Substance	Segment length (cm) and hair concentrations (pg/mg) from root to tip	Other positive matrices	
					Time between exposure and hair sampling (days)	Color	Hair bleaching	Number of analyzed segments			Blood (ng/mL)	Urine (ng/mL)
*Abuse in children*												
1.1	F	14	Pills	Drowsiness	390	Brown	Yes	13/13	Segment length	2.5–2.0–2.0–2.0–2.0–2.0–2.0^b^–2.0^b^–2.0^b^–2.0^b^–2.0^b^–2.0^b^–6.0	na	na
									Alprazolam	ND–ND–ND–ND–ND–ND–Traces^b^–Traces^b^–Traces^b^–0.81^b^–0.71^b^–1.97^b^–1.45		
1.2	F	8	Pills	Articular pains	390	Light	No	12/12	Segment length	2.5–2.0–2.0–2.0–2.0–2.0–2.0^b^–2.0^b^–2.0^b^–3.0^b^–4.0^b^–6.0	na	na
									Alprazolam	ND–ND–ND–ND–ND–ND–Traces^b^–Traces^b^–0.52^b^–0.63^b^–0.61^b^–Traces		
2	F	6	Pills	Drowsiness	42	Brown	No	12/12	Segment length	1.0–1.6^b^–1.7^b^–1.9–2.5–2.3–2.3–2.2–2.0–2.1–2.8–1.7	na	Positive benzodiazepine immunoassay
				Ataxia					Diazepam	1.5–5.7^b^–8.6^b^–4.9–5.2–5.2–5.2–6.5–8.3–9.4–11.5–18		
				Dysarthria								
*Abuse in the elderly*												
3	F	90	Drink	Drowsiness	0	Light	Yes	9/9	Segment length	1.0^b^–1.9^b^–1.0–1.0–1.0–2.0–2.0–1.9–2.0–3.5	Loprazolam (ND)	Loprazolam (133)
									Loprazolam	6.1^b^–3^b^–ND–ND–2.8–2.2–ND–ND–ND	Apixaban (nq)	Apixaban (nq)
									Tiapride	ND^b^–ND^b^–ND–ND–ND–554–35–ND–ND	Bisoprolol (nq)	Bisoprolol (ND)
											Furosemide (nq)	Furosemide (nq)
											Pantoprazole (nq)	Pantoprazole (nq)
4	F	86	Unknown	Unknown	19	Light	No	3/3	Segment length	2.0^b^–3.0^b^–3.5	Risperidone (4.25)	Risperidone (nq)
									Risperidone	54.9^b^–50^b^–11	Paroxetine (nq)	Paroxetine (nq)
									9‐OH‐rispéridone	49.7^b^–24.8^b^–4.5	Alprazolam (28)	Alprazolam (nq)
									Paroxetine	238^b^–196^b^–169	Mirtazapine (40)	Mirtazapine (nq)
									Alprazolam	20^b^–1.3^b^–ND	Bisoprolol (nq)	Bisoprolol
									Mirtazapine^a^	157^b^–41^b^–25	Warfarine (nq)	Warfarine
									DesmethylMirtazapine	133^b^–10–6.8	Furosemide (nq)	Furosemide
									Bisoprolol^a^	414^b^–319^b^–317		
5	H	77	Unknown	Asthenia	20	Light	No	2/2	Segment length	1.5^b^–2.5^b^	Alimemazine (nq)	Amlodipine (nq)
				Drowsiness					Alimemazine	108^b^–77^b^	Cyamemazine (nq)	Bisoprolol (nq)
				Dysarthria					Cyamemazine	125^b^–111^b^	Bisoprolol (nq)	Sotalol (nq)
				Lower limb weakness					Paroxetine	ND^b^–13^b^	Perindopril (nq)	Perindopril (nq)
									Hydroxyzine	8^b^–ND^b^	Levetiracetam metabolite (nq)	Oxazepam (nq)
									Oxazepam^a^	172^b^–224^b^		Topiramate (nq)
									Amitriptyline	32^b^–12^b^		Levetiracetam metabolite (nq)
									Nortriptyline	96^b^–10^b^		
									Bisoprolol^a^	357^b^–338^b^		

Abbreviations: na, not analyzed; ND, not detected; nq, not quantified.

^a^ Victim's treatment.

^b^ segment corresponding to alleged facts.

#### 3.3.1 Cases 1.1 and 1.2

Two siblings (aged 14 and 8) reported forced daily alprazolam administration by their mother, leading to excessive sleep. Hair analysis confirmed alprazolam presence in segments corresponding to the reported administration period, with no detection in subsequent samples after contact cessation. One child also reported episodic tramadol use, which ceased upon loss of maternal contact.

#### 3.3.2 Case 2

A 6‐year‐old child presented to the emergency room with drowsiness, dysarthria, ataxia, balance issues, and nystagmus. Urine analysis confirmed benzodiazepine presence. Hair collected 42 days later (12 brown segments analyzed) revealed diazepam in all segments. Concentrations ranged from 1.5 pg/mg (proximal segment) to 18 pg/mg (distal segment).

#### 3.3.3 Case 3

A 90‐year‐old woman, hospitalized for a femoral fracture, reported suspected chemical sedation occurring at home, characterized by episodes of drowsiness following the consumption of orange juice or tea. Blood, urine, and hair samples were collected three months after the onset of the alleged exposure. Hair analysis detected tiapride in two segments (554 pg/mg and 35 pg/mg) and loprazolam in four segments (2.2 pg/mg to 6.1 pg/mg), with the highest loprazolam concentrations observed in the proximal segments. Judicial investigation subsequently led to the daughter's conviction and a four‐year prison sentence.

#### 3.3.4 Case 4

An 86‐year‐old woman experienced recurrent episodes of drowsiness and hypotension during two hospitalizations within a one‐month period. Recurrence of somnolence was observed after a visitor provided a cake. Hair analysis identified risperidone and alprazolam, with the highest concentrations of both substances detected in the proximal hair segment. Initial and in‐hospital biological samples (blood and urine) confirmed the presence of the same substances identified in hair. Notably, only a risperidone metabolite was identified at admission, whereas risperidone itself was detected in subsequent in‐hospital samples. The patient's documented medication regimen included only mirtazapine and bisoprolol, with no prescription for risperidone or alprazolam during hospitalization.

#### 3.3.5 Case 5

A 77‐year‐old man was hospitalized on five occasions over a three‐month period, consistently presenting with dysarthria, severe asthenia, lower limb weakness, and marked drowsiness lasting approximately 24 h. Biological samples were collected within 24 h following the last suspected exposure. Among the substances detected in hair, only oxazepam and bisoprolol were consistent with the patient's prescribed medications. Cyamemazine, alimemazine, amitriptyline, paroxetine, and hydroxyzine were also detected but not part of the patient's reported treatment regimen. Cyamemazine and alimemazine were prescribed medications of an individual in the patient's social environment who was suspected of committing fraud against him.

### 3.4 Other cases

Seven additional positive cases from various forensic investigations are summarized in Table 5. These cases encompassed diverse circumstances, including a minor exhibiting high‐risk behavior, a criminal offender, suspected maternal transmission of substances to an infant via breastfeeding, a suspected needle‐spiking incident, a suspected administration of a harmful substance through a beverage, and two cases for which limited contextual information was available.

**TABLE 5:** Additional positive cases from forensic investigations involving diverse circumstances.

Case	Sex	Age (years)	Context	Symptoms	Hair analysis				Substance	Segment length (cm) and hair concentrations (pg/mg) from root to tip	Other positive matrices	
					Time between the facts and hair sampling (days)	Color	Hair bleaching	Number of analyzed segments			Blood (ng/mL)	Urine (ng/mL)
1	F	28	Unknown	Dizziness	42	Brown	No	5/5	Segment length	1.5^b^–2.0–2.0–3.0–3.5	na	na
				Psychic slowdown					MDMA	90.1^b^–35.2–49.3–60.9–91.7		
				Trismus					Codeine	11.7^b^–ND–ND–ND–ND		
				Bruxism								
				Muscle spasm								
2	F	22	Unknown	Amnesia	102	Brown	Yes	12/12 (+22 cm of unsegmented and unanalyzed tip)	Segment length	0.5–0.5–0.5–0.5–0.5–0.7–0.5^b^–0.5–0.7^c^–0.6–0.5–0.8	na	na
				Abdominal pain					Cocaine	ND–ND–ND–ND–ND–ND–ND^b^–99.6–131.3^c^–52.3–14–ND		
				Confusion					BZE	ND–ND–ND–ND–ND–ND–ND^b^–34.6–55.5^c^–21.6–ND–ND		
				Dizziness					MDMA	103.9–133.7–72.7–50.3–22.3–22.3–50.2^b^–45.3–77.3^c^–159.5–130.4–35.7		
				Tremors								
3	F	26	Criminal offender (murder)	–	15	Dark	Yes	8/8	Segment length	3.0^b^–3.1–2.9–3.0–3.0–3.0–4.0–11.0	na	BZE (34)
									MDMA	3720^b^–5280–1550–461–560–495–501–242		THC‐COOH (28)
									MDA	153^b^–206–80–53–63–52–56–ND		Methadone (7.4)
									Cocaine	120^b^–317–416–522–706–756–900–1160		EDDP (14)
									BZE	33^b^–95–121–143–216–276–407–289		
									THC	90^b^–90–109–133–128–110–207–95		
									Methadone	457^b^–324–984–1197–755–299–194–154		
									EDDP	27^b^–20–72–154–55–15–ND–ND		
									LSD	Traces^b^–ND in all others segments		
4	F	17	Minor with risky behavior (prostitution)	Unknown	Unknown	Brown	No	11/11	Segment Length	3.0–3.0–3.0–3.0–3.0–3.0 2.7–3.0–3.0–3.0–4.5	THC (1.3)	THC‐COOH (nq)
									Cocaine	48–182–363–360–333–300–495–242–250–164–211	THC‐COOH (11.6)	
									BZE	ND–23–52–56–40–30–32–34–37–27–44		
									THC	90–355–610–539–576–508–574–606–532–337–394		
									THC‐COOH	ND–1–2–2.4–3.4–3–3.7–3.5–3.3–2–2.5		
5	F	21	Child's exposure (suspected exposure through breastfeeding)	–	67	Dark	No	4/4	Segment length	1.5–2.0^b^–2.0–2.0	na	na
									Diazepam	ND–ND^b^–1.4–2.0		
									Zopiclone	2.2–5.7^b^–ND–ND–ND		
									Cocaine	56–192^b^–555–725		
									BZE	56–139^b^–358–553		
6	F	47	Suspicion of needle spiking	Asthenia	56	Dark	Yes	5/6	Segment length	1.0–1.0^b^–1.0–1.0–1.0	ND	ND
									Paroxetine^a^	804–416^b^–119–73–46		
7	F	27	Suspicion of a harmful substance being administered through a drink	Drowsiness	60	Dark	No	4/4 (+35 cm of unsegmented and unanalyzed tip)	Segment length	0.5^b^–1.0^b^–1.0–1.0	na	na
									Zolpidem	9.4^b^–ND^b^–ND–ND		

Abbreviations: na, not analyzed; ND, not detected; nq, not quantified.

^a^ Victim's treatment.

^b^ segment corresponding to alleged facts with a growth delay of 1 cm per month.

^c^ segment corresponding to alleged facts with a growth delay of 1.3 cm per month.

#### 3.4.1 Case 1

A 28‐year‐old female reported unexplained symptoms including dizziness, slow‐motion images, heavy limbs, trismus, bruxism, and muscle spasms after attending a nightclub. She denied habitual drug use or medication. Hair analysis detected MDMA and codeine in the segment corresponding to the reported events.

#### 3.4.2 Case 2

A 22‐year‐old female reported confusion, dizziness, amnesia, tremors, and abdominal pain following a party where four individuals experienced fainting and memory loss. She denied habitual drug use or medication. High concentrations of MDMA and cocaine and its metabolite were detected in the hair segment corresponding to the period of the reported events, based on a hair growth of 1.3 cm per month.

#### 3.4.3 Case 3

A 26‐year‐old woman under judicial investigation for homicide underwent urine and hair sampling two weeks after the alleged events. Her medical history included documented polysubstance use, including cannabis, cocaine, amphetamines, and lysergic acid diethylamide (LSD).

#### 3.4.4 Case 4

A 17‐year‐old minor presenting high‐risk behaviors, including repeated runaway episodes, engagement in prostitution, and uncontrolled substance use, was investigated in a forensic context.

#### 3.4.5 Case 5

A 21‐year‐old woman underwent hair sampling as part of a forensic investigation following the death of her 7‐month‐old infant. Approximately one month before death, the infant was hospitalized for abrupt behavioral changes, including hypersomnolence and ptosis, and urine analysis revealed diazepam and its three metabolites. Mixed feeding (breastfeeding and expressed breast milk) was reported. No hair samples were collected from the infant, and no medications or illicit substances were detected in post‐mortem samples. Maternal hair analysis, performed approximately two months and one week after the alleged events, showed no concordance with the substances identified in the infant's urine.

#### 3.4.6 Case 6

A 47‐year‐old woman reported suspected needle spiking while in a shopping center. She described sensing a cold liquid on her thigh and subsequently observed a hole and a blood stain on her trousers, followed by asthenia. During the forensic examination, a millimetric puncture lesion was observed on the affected thigh. No medicinal or toxic substances were detected in blood or urine samples collected approximately 26 h after the incident, nor in hair samples collected approximately 8 weeks later, apart from the victim's prescribed treatment (paroxetine 10 mg/day), which was identified only in hair.

#### 3.4.7 Case 7

A 27‐year‐old woman reported spending an evening in a bar, during which she consumed a drink that had been left unattended for a period of time. She subsequently experienced marked somnolence, requiring her partner to be contacted to escort her home. A second similar episode was reported, raising suspicion of attempted DFC. Hair samples were collected two weeks after the most recent suspected exposure.

## 4 DISCUSSION

### 4.1 Children exposure

Four cases involving children and their families and two additional cases involving only children were analyzed in this study.

Children spend a significant amount of time in close proximity to their parents within the home environment and are particularly vulnerable to environmental exposure. As mobility develops, exploratory behavior, such as touching surfaces and placing objects in their mouths, increases the risk of contact with contaminated substances. Furthermore, hair characteristics differ between children and adults and may influence the incorporation and retention of toxic substances depending on age and the sampling period. Several studies have demonstrated that children's hair is typically thinner and more porous than adult hair, facilitating both external incorporation of substances and potentially enhanced elimination through washing practices such as shampooing [17, 18, 19]. This latter characteristic may partly explain the absence of certain molecules, such as methadone or other psychoactive drugs, in our pediatric cases, in contrast to numerous reports in the literature [17].

In this series, cocaine and its metabolite BZE, together with THC, were the main substances detected in cases of child exposure, in agreement with previous studies [20, 21]. Accidental cocaine intoxication in young children has been widely reported and is typically associated with symptoms such as tachycardia, seizures, agitation, and altered consciousness [8, 22, 23, 24]. Although Arbouche et al. [21] reported drowsiness as the most frequent symptom in pediatric cannabis intoxications, this was not observed in our cohort, even in cases with positive results in other biological matrices. The absence of a clear correlation between toxicological results and clinical symptoms in our study supports environmental exposure rather than acute accidental poisoning. This observation is consistent with a 2023 Italian study [8] reporting no statistically significant association between substance concentrations and clinical manifestations, suggesting that exposure levels may not directly predict clinical severity. In addition, the potential for missing or incomplete data must be considered. Case 2.1 is particularly noteworthy, as the simultaneous detection of cocaine and BZE in hair, urine, and blood strongly suggested active exposure (Table 3). The concentration ratio between the first two proximal segments was close to 1:2, and concentrations measured in the child were approximately tenfold higher than those observed in the aunt. Overall, cocaine and THC concentrations tended to be higher in younger children, with ratios ranging from 10 to 100 compared with adults, a finding consistent with previous reports and likely related to age‐dependent hair characteristics [25].

Substance concentrations in the suspected adult consumers generally exceeded the cut‐off values for drugs of abuse proposed by the 2021 Santiago consensus of the Society of Hair Testing (SoHT) [12], with the exception of the father in Case 4.4 (Table 3). It should be noted that these cut‐offs are validated for head hair only [11]. Axillary hair is not the best alternative because of potential wash‐out effect from sweat, differences in drug incorporation mechanisms, and generally lower concentrations reported for many compounds [26]. Nevertheless, axillary hair analysis, together with other keratinized matrices such as nails when head hair is unavailable [27, 28], remains valuable for qualitative assessment. Moreover, analysis of all household members, including fathers who may be less accessible as highlighted in previous studies [25], is essential for accurately documenting each family member's exposure or consumption histories.

THC‐COOH, a metabolite indicative of active THC use, was not detected in the hair of any suspected adult users in these cases and was found in the urine of only one mother. In contrast, THC‐COOH was detected in both blood and hair in Case 6 (Table 3), suggesting very recent intentional consumption by the child shortly before sample collection. These findings may also be related to the relatively high LLOQ (1 pg/mg), whereas the minimum required LOQ for THC‐COOH confirmation is 0.2 pg/mg [12].

These cases highlight the difficulties in determining the precise source of drug exposure in children. Potential pathways include in‐utero transfer, postnatal exposure through breastfeeding, environmental contamination via inhalation or ingestion (accidental or intentional, such as in Munchausen syndrome by proxy), and external deposition onto hair from passive smoke, dust, contaminated surfaces, or parental sebum and sweat [5, 6, 29, 30, 31, 32]. This complexity is further compounded by the difficulty in distinguishing acute from chronic exposure in pediatric populations, an issue emphasized by Alvarez et al., who recommend caution in the interpretation of results, particularly in children younger than three years [33].

### 4.2 Chemical abuse in children

This study documented three cases of suspected chronic chemical abuse in children: one involving alprazolam in two girls and another involving diazepam in a single girl. Intentional and repeated administration of drugs in the context of child abuse is considered rare and is likely underreported, in part due to difficulties in clinical recognition and diagnosis. Reported cases typically involve very young children, often around two years of age, frequently implicate the mother, and are commonly associated with Munchausen syndrome by proxy [34]. Anticonvulsants are among the most frequently identified substances in these situations [7, 35].

As highlighted in the literature, interpretation of these limited data remains challenging because of the small number of documented cases and the absence of a control group. However, the identification of specific substances may strongly suggest abuse, particularly when prescription guidelines based on age are violated. In this context, the detection of alprazolam, a medication generally restricted to individuals over 18 years of age [36], constitutes compelling evidence suggestive of intentional exposure.

### 4.3 Elderly: Medications versus abuse

Three cases involving individuals older than 75 years were identified in this study. One case was documented as chemical abuse, with the detection of loprazolam and tiapride. The loprazolam concentrations observed were comparable to those reported by Kintz et al., whereas tiapride concentrations were higher than those previously described in three cases of intoxication, which ranged from 13 to 148 pg/mg [9]. A second case, for which no clear motive was identified, showed the presence of risperidone, paroxetine, and alprazolam, none of which were part of the individual's documented medication regimen. A third case, associated with suspected fraud, revealed the presence of alimemazine, cyamemazine, hydroxyzine, and amitriptyline.

Although melanin affinity is a major factor influencing the incorporation of basic drugs into hair, the detection of substances in hair segments corresponding to the alleged exposure suggests that light or bleached hair did not substantially affect the interpretability of our results. These findings support the utility of hair sampling as a valuable matrix for detecting chemical abuse in elderly individuals, particularly in cases of repeated or long‐term exposure.

In addition to its extended detection window, hair analysis enables the identification of repeated drug administration through segmental analysis, thereby facilitating differentiation between chronic maltreatment and isolated accidental exposure [37]. Although none of the cases reported here resulted in death, hair analysis may be crucial in fatal cases for elucidating long‐term or chronic intoxication, as well as instances of neglect involving failure to administer prescribed medication, even in post‐mortem contexts [9, 38, 39]. The prevalence of chemical administration in elderly individuals is likely underestimated. Indeed, a French national study reported that approximately 3% of chemical submission cases involved victims over 70 years of age [2].

### 4.4 Other cases

The detection of multiple substances in hair samples significantly broadens the potential applications of this analytical approach beyond the scope of domestic child exposure. Hair analysis may also be a valuable tool for screening drug use in diverse forensic contexts, including investigations involving suspected offenders and the assessment of high‐risk behaviors in minors. In this study, one case involved suspected needle spiking in a young woman, with negative analytical findings supporting the results of a retrospective study conducted in the forensic medicine department of Paris in 2022 [40].

However, the wider implementation of these applications depends on the establishment of robust reference values across a broader range of substances and age groups. Although our analytical method included 166 NPS, as recommended by the French Society of Analytical Toxicology [41], none were detected in any of the cases, including those in which MDMA or LSD were identified. This observation is consistent with previous findings indicating a relatively low prevalence of NPS detection in hair samples from individuals diagnosed with substance use disorders [42]. Beyond psychoactive substances, hair analysis has also been reported as a useful tool for detecting compounds administered for other purposes, such as substances used to induce abortion [3].

## 5 CONCLUSION

This study presents the results of a six‐year investigation of hair drug analyses conducted in the context of DFC, involving both victims and suspected perpetrators. Hair concentrations from 26 positive cases are reported, encompassing a wide range of ages, hair characteristics, exposure timeframes, and patterns of single or repeated exposure to various psychoactive substances across diverse circumstances, including child exposure, chemical abuse in children, and abuse in elderly individuals.

This work provides valuable comparative data to support the interpretation of hair toxicology findings, particularly in vulnerable populations. Such cases are especially challenging because of the complexity of exposure pathways, limitations of available reference values, and the significant forensic implications associated with result interpretation.

## CONFLICT OF INTEREST STATEMENT

The authors have no conflicts of interest to declare.

## Supporting information


**TABLE S1.** MRM transitions, voltage settings declustering potential (DP), entrance potential (EP), collision energy (CE), and collision cell exit potential (CXP) for each analyte of the MS/MS methods on API5500 QTRAP mass spectrometer.

**TABLE S2.** Validation data for the main drugs searched in hair sample.

## Data Availability

The data that support the findings of this study are available from the corresponding author upon reasonable request.
