# Supplementary material for: Insights from a six‐year hair drug analysis compendium in drug‐facilitated crimes involving vulnerable population cases
Source: J Forensic Sci. 2026 Apr 16;71(4):1767–82. doi: 10.1111/1556-4029.70330 (PMC13340945; doi:10.1111/1556-4029.70330)
Supplement: Supplementary file 2 — TABLE S2. Validation data for the main drugs searched in hair sample. [file JFO-71-1767-s001.docx]

TABLE S2 Validation data for the main drugs searched in hair sample.

|  | **Lower limit of detection**  **(pg/mg)** | **Lower limit of quantification (pg/mg)** |
| --- | --- | --- |
| **Benzodiazepines** |  |  |
| 7-aminoclonazepam | 2.5 | 5 |
| 7-aminoflunitrazepam | 2.5 | 5 |
| alprazolam | 0.5 | 1 |
| bromazepam | 2.5 | 5 |
| clobazam | 1 | 2.5 |
| clonazepam | 1 | 2.5 |
| desmethylclobazam | 1 | 2.5 |
| desmethylflunitrazepam | 1 | 2.5 |
| diazepam | 1 | 2.5 |
| estazolam | 1 | 2.5 |
| flunitrazepam | 0.5 | 1 |
| hydroxyalprazolam | 1 | 2.5 |
| hydroxyflunitrazepam | 2.5 | 5 |
| hydroxymidazolam | 2.5 | 5 |
| ethyl loflazepate | 1 | 2.5 |
| loprazolam | 1 | 2.5 |
| lorazepam | 5 | 10 |
| lormetazepam | 2.5 | 5 |
| midazolam | 1 | 2.5 |
| nitrazepam | 1 | 2.5 |
| nordiazepam | 2.5 | 5 |
| oxazepam | 5 | 10 |
| prazepam | 1 | 2.5 |
| temazepam | 1 | 2.5 |
| tetrazepam | 2.5 | 5 |
| triazolam | 1 | 2.5 |
| zolpidem | 0.5 | 1 |
| zopiclone | 0.5 | 1 |
| **Sedatives** |  |  |
| alimemazine | 0.5 | 1 |
| amitriptyline | 0.5 | 1 |
| amoxapine | 0.5 | 1 |
| atropine | 2.5 | 5 |
| brompheniramine | 1 | 2.5 |
| cetirizine | 1 | 2.5 |
| chlorpheniramine | 1 | 2.5 |
| chlorpromazine | 1 | 2.5 |
| clozapine | 1 | 2.5 |
| cyamemazine | 0.5 | 1 |
| diphenydramine | 1 | 2.5 |
| doxylamine | 0.5 | 1 |
| haloperidol | 0.5 | 1 |
| hydroxyzine | 0.5 | 1 |
| ketamine | 5 | 10 |
| levomepromazine | 0.5 | 1 |
| loratadine | 2.5 | 5 |
| loxapine | 0.5 | 1 |
| mirtazapine | 0.5 | 0.5 |
| niaprazine | 0.5 | 1 |
| norketamine | 5 | 10 |
| normirtazapine | 1 | 1 |
| o-desmethyltramadol | 1 | 2.5 |
| paliperidone | 0.5 | 1 |
| paroxetine | 2.5 | 5 |
| pheniramine | 1 | 2.5 |
| promethazine | 2.5 | 5 |
| risperidone | 0.5 | 1 |
| scopolamine | 1 | 2.5 |
| tiapride | 1 | 1 |
| tramadol | 1 | 2.5 |
| trihehyphenidyl | 0.5 | 1 |
| tropatepine | 1 | 2.5 |
| **Narcotics** |  |  |
| amphetamine | 10 | 10 |
| methamphetamine | 10 | 10 |
| MDMA | 10 | 10 |
| MDA | 10 | 10 |
| MDEA | 10 | 10 |
| cocaine | 10 | 10 |
| benzoylecgonine | 10 | 10 |
| ecgonine methyl ester | 10 | 10 |
| cocaethylene | 10 | 10 |
| norcocaine | 10 | 10 |
| morphine | 10 | 10 |
| 6-MAM | 10 | 10 |
| codeine | 10 | 10 |
| ethylmorphine | 10 | 10 |
| dihydrocodéine | 10 | 10 |
| pholcodine | 10 | 10 |
| oxycodone | 10 | 10 |
| methadone | 10 | 10 |
| EDDP | 10 | 10 |
| THC | 1 | 5 |
| CBD | 10 | 10 |
| CBN | 30 | 30 |
| THC-COOH | 1 | 1 |
| LSD | 1 | 1 |
